# Supplementary material for: Complexome profiling of the Chlamydomonas psb28 mutant reveals TEF5 as an early PSII assembly factor
Source: Plant Cell. 2025 Mar 17;37(6):koaf055. doi: 10.1093/plcell/koaf055 (PMC12164590; doi:10.1093/plcell/koaf055)
Supplement: koaf055_Supplementary_Data [file koaf055_supplementary_data.zip › psb28_interactive.zip!/plots/intensity/qualityPlots/peptides/_Cre03.g180000.t1.2.html]

### Cre03.g180000.t1.2

Trivial name:   
SeqCoverage [median]: 9.10  
Mass: 34.12 kDa  
  
 

| Sequence | MissedCleavage | Mass [Da] | wtIntensity | muIntensity | Score |
| --- | --- | --- | --- | --- | --- |
| ALLEEVAER | 0 | 1028.55 | 0.00 | 1155.92 | 94.30 |
| LTDMGHAPIR | 0 | 1109.57 | 0.00 | 352.44 | 46.82 |
| TAAEQVAEFVR | 0 | 1219.62 | 0.00 | 2357.25 | 94.69 |
|  |  |  | 0.0 | 3865.6 |  |
